# Morphology of the Cutaneous Poison and Mucous Glands in Amphibians with Particular Emphasis on Caecilians (*Siphonops annulatus*)

**DOI:** 10.3390/toxins13110779

**Published:** 2021-11-04

**Authors:** Beatriz Mauricio, Pedro Luiz Mailho-Fontana, Luciana Almeida Sato, Flavia Ferreira Barbosa, Renato Mancini Astray, Alexander Kupfer, Edmund D. Brodie, Carlos Jared, Marta Maria Antoniazzi

**Affiliations:** 1 Laboratory of Structural Biology, Instituto Butantan, São Paulo 05509-000, Brazil; beatriz.mauricio@butantan.gov.br (B.M.); pedro.fontana@esib.butantan.gov.br (P.L.M.-F.); sato.lucy@gmail.com (L.A.S.); marta.antoniazzi@butantan.gov.br (M.M.A.); 2 Multipurpose Laboratory, Instituto Butantan, São Paulo 05503-000, Brazil; flavia.barbosa@butantan.gov.br (F.F.B.); renato.astray@butantan.gov.br (R.M.A.); 3 Department of Zoology, State Museum of Natural History, 70191 Stuttgart, Germany; alexander.kupfer@smns-bw.de; 4 Department of Biology, Utah State University, Logan, UT 84322, USA; e.brodie@usu.edu

**Keywords:** amphibia, Gymnophiona, skin glands, poison, toxins

## Abstract

Caecilians (order Gymnophiona) are apodan, snake-like amphibians, usually with fossorial habits, constituting one of the most unknown groups of terrestrial vertebrates. As in orders Anura (frogs, tree frogs and toads) and Caudata (salamanders and newts), the caecilian skin is rich in mucous glands, responsible for body lubrication, and poison glands, producing varied toxins used in defence against predators and microorganisms. Whereas in anurans and caudatans skin gland morphology has been well studied, caecilian poison glands remain poorly elucidated. Here we characterised the skin gland morphology of the caecilian *Siphonops annulatus*, emphasising the poison glands in comparison to those of anurans and salamanders. We showed that *S. annulatus* glands are similar to those of salamanders, consisting of several syncytial compartments full of granules composed of protein material but showing some differentiated apical compartments containing mucus. An unusual structure resembling a mucous gland is frequently observed in lateral/apical position, apparently connected to the main duct. We conclude that the morphology of skin poison glands in caecilians is more similar to salamander glands when compared to anuran glands that show a much-simplified structure.

## 1. Introduction

Among amphibians, the skin performs, in addition to physical protection, several other vital functions such as gas exchange, ion and water transport, temperature control, chemical and mechanical sensory reception, reproduction and defence against predators and microorganisms [1,2,3,4,5]. In many of these functions, skin glands of two types located in the dermis, the mucous glands and the poison glands, which are characteristic of all amphibian orders [3,4,5], play a fundamental role [1,3,6].

Amphibians belonging to the order Gymnophiona, commonly referred to as caecilians, are vertebrates primarily adapted to a fossorial life [3]. They constitute a unique group within the class Amphibia showing, among several characteristics, a limbless and ringed body. They also show reduced eyes, a pair of sensory tentacles, and an intromittent copulatory organ [7,8]. In addition, many oviparous species have a unique type of parental care, known as “skin feeding” (or dermatophagy), in which the young feed on the maternal skin during their development [9,10,11,12]. With only 214 species [13], these animals constitute the least known group of Class Amphibia [11,14,15]. Essentially with tropical distribution, they are relatively abundant in South America, where the species *Siphonops annulatus* is widely distributed and adapted to different humidity levels ranging from dry (semi-arid) regions to tropical forests [16].

The skin of caecilians is very well adapted to a fossorial lifestyle, including the external homogeneity of the moist and slippery body surface facilitating movement in the soil [17]. The skin does not present external apparent glandular accumulations, such as the parotoid macroglands of toads, certain tree frogs and salamanders, used in defence against predators [4,17,18,19,20,21]. However, in *Siphonops annulatus*, and presumably in all other caecilians, the skin glands show a polarised distribution, with mucous glands predominating in the head, and the poison glands being much larger and numerous in the tail region [17,22]. Anuran skin glands were quite well characterised in numerous morphological studies [1,18,23,24] and conversely the cutaneous glands of caecilian amphibians remain very little studied. A previous study on the subject showed that the morphology of the poison glands in these amphibians is quite complex and deserves more accurate examination [17]. The morphological organisation and functioning mechanism of such glands show similarities with salamanders [25,26,27] and, are quite different from those of anurans [1,18,24,28,29]. Likewise, information about the chemical composition and biological action of caecilian skin secretion is scarce. In *S. annulatus*, although the poison glands have been associated with a defensive role for almost a century [22,30], until the 1970s, there was no evidence that they could produce real toxins. They were only associated with haemolytic and cardiotoxic action [31,32] and, more recently, with antiparasitic activity [33].

Given so many open questions, the present study aims to complement and extend our previous findings about *Siphonops annulatus* skin [17], going into details of its morphology and histochemistry. We give particular emphasis to the unusual structure of the poison glands, revealing new features and proposing possible functioning mechanisms of the secretory process. Finally, we compare and correlate the results with those already available for species of the orders Anura and Caudata.

## 2. Results

### 2.1. General Characteristics of the Skin

**Figure 1:**
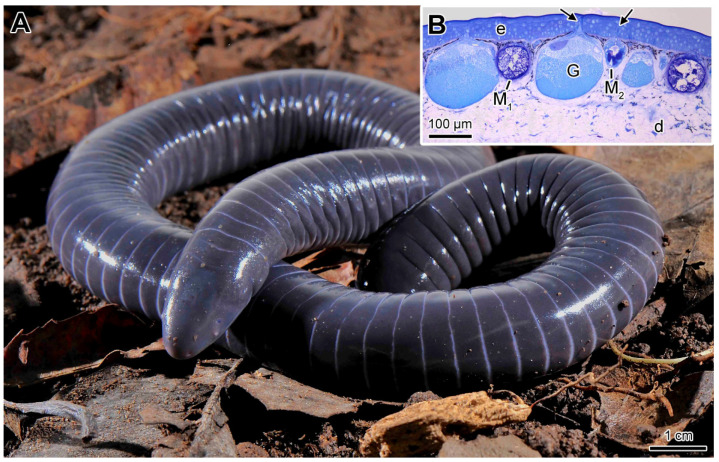
(**A**) *Siphonops annulatus.* (**B**) Histological section showing the general appearance of the skin. (arrows) ducts, (e) epidermis, (d) dermis, (G) granular gland, (M_1_ and M_2_) mucous glands. Staining: toluidine blue-fuchsin.

The histological analysis of *Siphonops annulatus* (Figure 1A) skin showed that the epidermis varies in thickness according to the region of the body and is thicker on the back (80–100 µm) when compared to the ventral face (60–70 µm).

The outermost portion of the dermis, the spongy layer, houses many multicellular glands of acinar shape (Figure 1B), externally surrounded by a monolayer of myoepithelial cells and are connected to the surface by an epithelial duct. Two different types of cutaneous gland were observed: the mucous glands and the poison (or granular) glands, distinguishable by their shape, size, cell organization, existence or absence of a lumen, and morphological characteristics of the secretion (Figure 1B). This set of defining characters is already well established in literature especially for anurans [1,3], but also for salamanders [25,26,27,34] and a few caecilians [35,36] including a previous, more superficial article on *Siphonops annulatus* conducted by our group [17]. Just below the glandular layer, the dermis forms a compact layer, mainly consisting of collagen fibres.

The glands were examined in detail, according to the description in the following sections, and no apparent histological and ultrastructural differences were observed along the body, except for gland variation in number and size, subject that was already treated in our previous paper [17]. For this reason, the results, unless explained, refer to the mid-body region.

### 2.2. Mucous Glands

The mucous glands are formed by a monolayer of secretory cells delimiting a central lumen (Figure 1B). The secretory cells have basal nuclei, and cytoplasm full of secretion granules. In the skin of *Siphonops annulatus,* two distinct types of mucous gland are recognized, here denominated M_1_ and M_2_ glands (Figure 1B).

**Figure 2:**
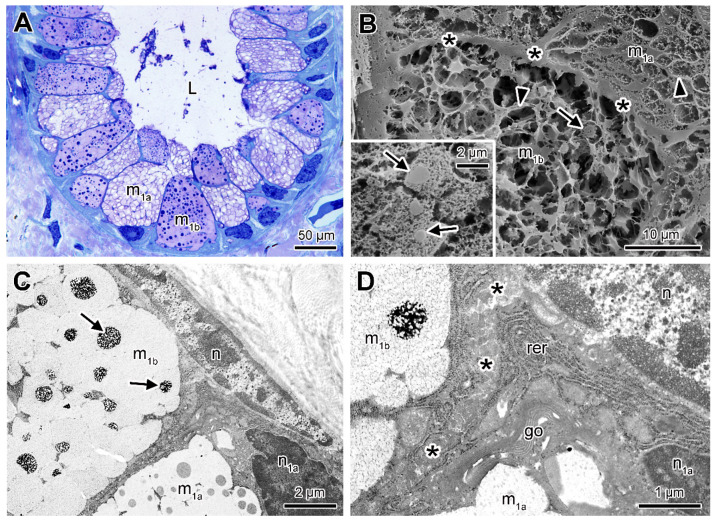
Mucous gland type 1 containing two cell types (m_1a_ and m_1b_). (**A**) Histological cross-section of the skin showing the cells (m_1a_ and m_1b_) with granules showing different affinities to toluidine blue-fuchsin. (**B**) SEM showing the morphology of the secretion granules of the different cells (m_1a_ and m_1b_). The arrowhead indicates the boundaries between the granules and the arrows indicate the dense cores within many of the granules of the m_1b_ cells. The insert indicates medium density cores in m_1a_ cell. (**C**) TEM showing part of two neighbouring secretory cells (m_1a_ and m_1b_), filled with aggregated granules. Note the flocculent electron dense cores of the granules of the m_1b_ cell (arrows). (**D**) Periphery of the secretory cells with cytoplasm showing rough endoplasmic reticulum (rer) and Golgi apparatus (go). (L) lumen, (n) nucleus of a myoepithelial cell, (n_1a_) nucleus of m_1a_ cell, (*) cell limits.

The M_1_ glands are larger and oval-shaped, composed of two types of secretory cells (m_1a_ and m_1b_) (Figure 2A). Both cell types have a prismatic form, and the cytoplasm is mainly filled by rounded and juxtaposed granules with moderate affinity to toluidine blue (Figure 2A). While the granules of m_1a_ cells are individualized and lightly stained, the granules of m_1b_ cells are more homogeneous and contain numerous rounded spots that are highly stained (Figure 2A). Scanning electron microscopy (SEM) of gland fractures show the sharp limits between the cells attesting their polygonal shape. While m_1a_ cells show granules with loose appearance revealing the presence of homogeneous dense cores (not visible by histology), m_1b_ cells show granules with hollow appearance (Figure 2B), in addition to the evident cores that were already identified in histological sections. Under transmission electron microscopy (TEM), the granules of both cells m_1a_ and m_1b_ appear electron lucent and flocculent and form a single mass of secretion due to their high level of coalescence (Figure 2C). In addition, the spherical cores of the m_1a_ cells show lower electron density when compared to those of the m_1b_ cells. At the base of both cell types, besides the nuclei of irregular shape, the cytoplasm is rich in organelles such as the rough endoplasmic reticulum and Golgi apparatus (Figure 2D).

**Figure 3:**
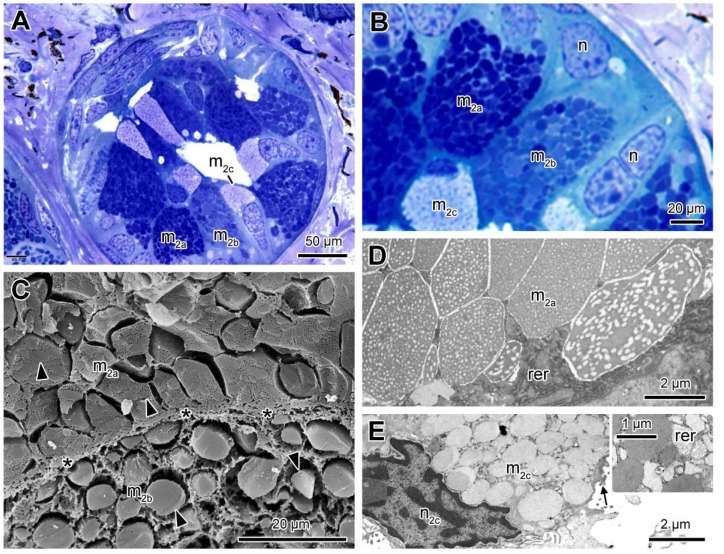
Mucous gland type 2 (M_2_) containing three cell types (m_2a_, m_2b_ and m_2c_). (**A**) Histological section showing cells with different affinities to toluidine blue-fuchsin. (**B**) High magnification of the cells and the different types of secretion granules. (**C**) Scanning electron microscopy (SEM) fracture showing m_2a_ cell, with polygonal granules, next to a m_2b_ cell, with round or oval granules. Note the difference in texture between the granules of the two cells (arrowheads). (**D**) TEM of part of a m_2a_ cell, focusing the substructure of the granules. (**E**) Transmission electron microscopy (TEM) image of part of a m_2c_ cell, with granules of smaller dimensions, lower electron density, and homogeneous texture. The insert shows the rough endoplasmic reticulum (rer) among the granules. (n) nucleus, (n_2c_) nucleus of m_2c_ cell, (arrow) microvilli, (*) cell limits.

The M_2_ glands are smaller when compared to the M_1_ glands. They are composed of three cell types (m_2a_, m_2b_ and m_2c_) (Figure 3A). The m_2a_ cells are the most abundant, with a cytoplasm filled with granules with intense affinity to toluidine blue (Figure 3A). Cells of the m_2b_ type are similar to m_2a_ cells in terms of shape and size but differing by the heterogeneous affinity of the secretion granules to toluidine blue (Figure 3B). Cells of the m_2c_ type are sparser and show smaller dimensions when compared to the other cell types (Figure 3A), with an elongate nucleus and smaller rounded granules, showing low affinity to toluidine blue (Figure 3A,B). At SEM the granules of m_2a_ cells appear juxtaposed and show a polygonal shape, presenting a matrix with a lacy aspect (Figure 3C). In m_2b_ cells, the granules have a rounded or oval shape, with varied sizes and homogeneous texture, and are immersed in a loose cytoplasm matrix (Figure 3C). With TEM, the granules of m_2a_ cells exhibit a sub granular texture matching with SEM images (Figure 3D). At the periphery a well-developed rough endoplasmic reticulum is observed. The m_2c_ cells show rounded and homogeneous granules of smaller dimensions and moderate electron density when compared to the other cell types (Figure 3E). Within the granules, a well-developed rough endoplasmic reticulum is observed. The granules are released into the lumen in the apical region in between microvilli (Figure 3E). The m_2b_ cells were not recognized by electron microscopy.

**Figure 4:**
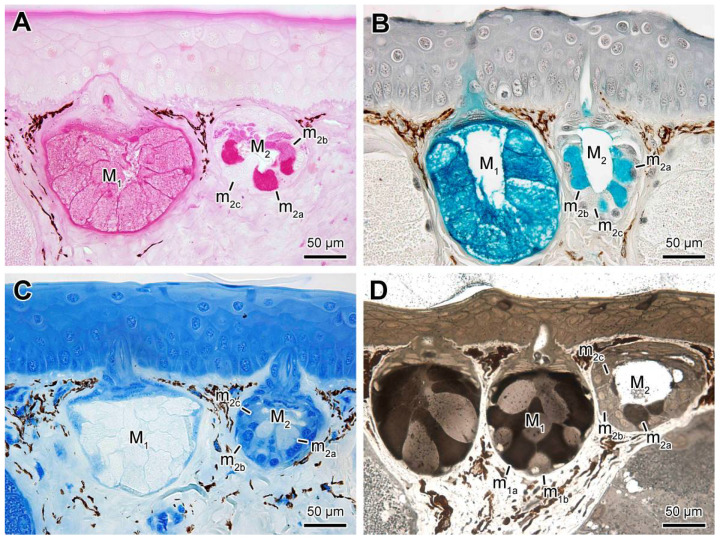
Histochemistry performed on the skin of *Siphonops annulatus* showing the mucous glands (M_1_ and M_2_) and their different cells (m_1a_, m_1b_, and m_2a_, m_2b_, m_2c_, respectively). (**A**) PAS (periodic acid-Schiff) indicative of neutral mucopolysaccharides. (**B**) Alcian blue, pH 2.5, indicating acid mucopolysaccharides. (**C**) Bromophenol blue, indicative of protein content. (**D**) Sudan black B, indicating lipids.

Regarding the applied histochemical methods, both cell types of the M_1_ glands were positive to PAS (periodic acid-Schiff) and alcian blue pH2.5 (Figure 4A,B), revealing neutral and acid mucopolysaccharides, respectively. Concerning protein content, the bromophenol blue reaction revealed that both cells were negative (Figure 4C). On the other hand, only m_1a_ cells were intensely reactive to Sudan black (Figure 4D), revealing the presence of lipids. In M_2_ glands, m_2a_ cells, besides being positive to PAS (Figure 4A) and, alcian blue pH 2.5 (Figure 4B), they were also positive to Sudan black (Figure 4D). However, the histochemical composition of m_2b_ cells was similar to the m_2a_ cells, differing only by the absence of reaction to Sudan black (Figure 4A–D), indicative of the absence of lipid content. Cells of m_2c_ type were reactive only to bromophenol blue (Figure 4C) indicating that most cell content is of protein nature.

### 2.3. Poison Glands

**Figure 5:**
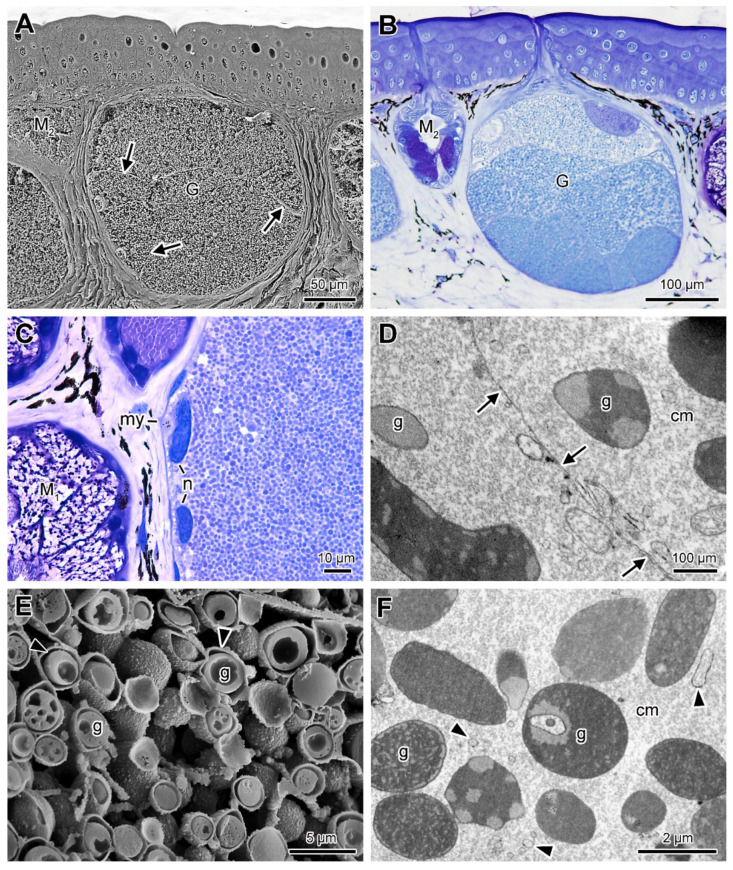
Skin poison glands (G) of *Siphonops annulatus*. (**A**) SEM image, showing that the glands are divided into syncytial compartments. The arrows point to the syncytia boundaries. (**B**) Histological section of the posterior region of the head, showing a round granular gland (G), with granules with affinity to toluidine blue. (**C**) High magnification of a histological section showing part of a poison gland on the right, with the peripheral nuclei of the syncytium (n), great amount of secretion granules, and the surrounding myoepithelial layer (my). (**D**) TEM showing the smooth limit between the membranes of two syncytial compartments (arrows), poor in interdigitations. (**E**) SEM fracture showing the secretion granules (g). Note the external layer (arrowheads) and the heterogeneous internal aspect. (**F**) TEM of part of the syncytial compartment showing heterogeneity both in shape and internal pattern of the granules (g), with different levels of electron density. Note the numerous dispersed vesicles (arrowheads). (M_1_, M_2_) mucous glands type 1 and 2, respectively; (cm) cytoplasm matrix.

The poison glands of *Siphonops annulatus* are always larger than the mucous glands (Figure 1B or Figure 5A,B). They vary in shape and size according to the region of the body and are more abundant in the tail, where they have the largest dimensions, occupying practically the entire dermis.

**Figure 6:**
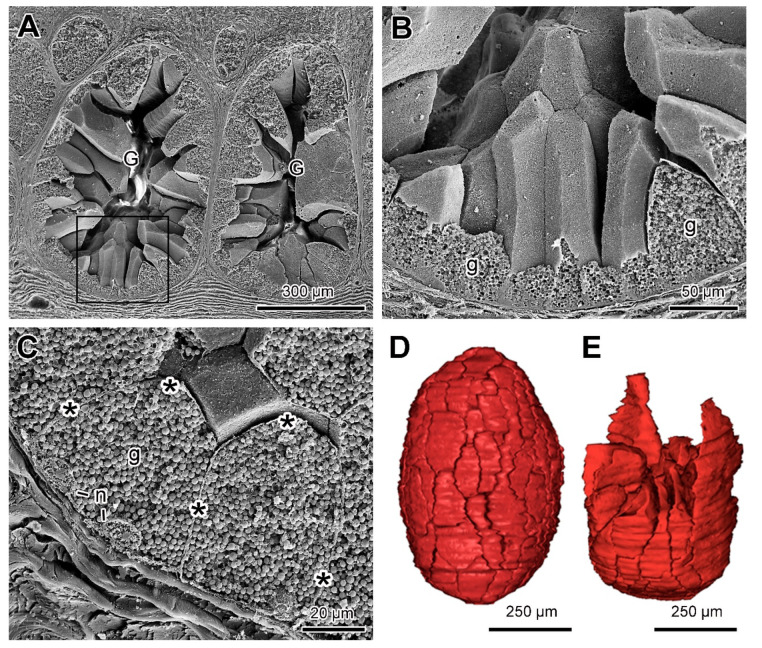
SEM and 3D reconstruction of the poison glands of *Siphonops annulatus*. (**A**) longitudinal fracture of the granular glands (G) exposing the syncytial compartments. (**B**) Higher magnification of the region delimited in (A), emphasizing the polyhedral shape of the syncytial compartments that are filled with granules (g). (**C**) Higher magnification of the syncytial compartments showing the boundaries among them (*), the internal granules (g) and the basal nuclei (n). (**D**,**E**) Three-dimensional reconstruction of the poison gland obtained from aligned histological sections (Video S1). In (D), view of the entire gland, with the syncytial compartments fitting together. In (E), inferior part of the gland showing the perfect fit of the cells as in a puzzle.

Unlike the mucous glands, the poison glands do not have a lumen (Figure 5A,B). They are formed by several cellular compartments filled with small and spherical granules with different degrees of affinity to toluidine blue (Figure 5B). Generally, the highest affinity is observed in the most basal compartments (Figure 5B). Each cell compartment has at least two nuclei distributed in the periphery, forming, therefore, a syncytium (Figure 5C). The syncytial compartments do not appear to be firmly adhered to each other, as they do not show interdigitations or other types of cell junctions along their limiting membranes (Figure 5D or Figure 6). At TEM and SEM, the syncytial compartments show secretion granules with heterogeneous shapes and different levels of electron density, forming internal patterns immersed in electron lucent cytoplasm containing numerous vesicles among the secretion granules (Figure 5D–F).

Cryo-fractures of the granular gland examined by SEM evidenced the polyhedral shape and the varied sizes of each syncytial compartment, delimited by smooth membranes, and virtually deprived of membrane junctions (Figure 6A–E). Fractures of the syncytial compartments show the nuclei located at the periphery, while the rest of the cytoplasm is filled with secretion granules (Figure 6B,C).

Three-dimensional reconstructions showed that, depending on the size of the glands, there was variation in the number of syncytial compartments (Video S1). Large glands such as those in the tail can reach up to 90 compartments. Once more the lack of cohesion between the compartments was confirmed. The results of SEM fractures, together with 3D reconstructions, demonstrated that all compartments fit together, similarly to pieces of a puzzle, generating the three-dimensional structure of the poison gland (Figure 6D,E).

**Figure 7:**
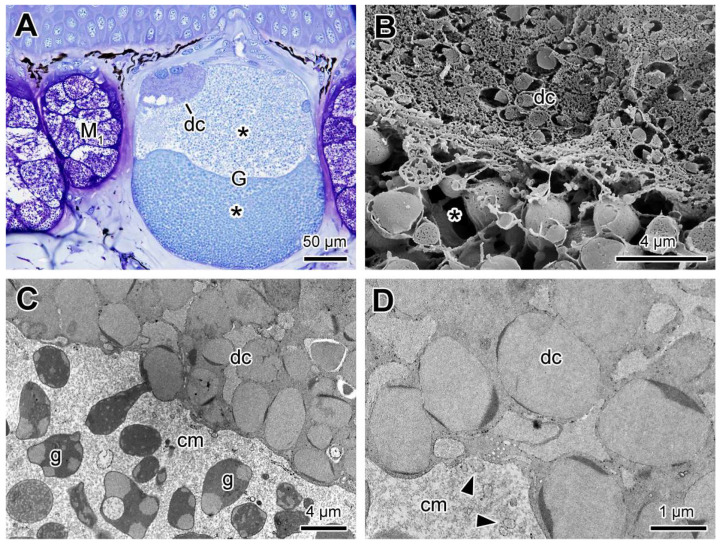
(**A**) Differentiated syncytial compartments in the poison gland of *Siphonops annulatus*. (**A**) Histological section showing a poison gland (G) in which a differentiated compartment (dc) is seen in the apical region, with small granules and two nuclei on the periphery, in addition to the common syncytial compartments (*). (**B**) SEM fracture comparing the granules of the apical differentiated compartment (dc) and of the common syncytial compartments (*). (**C**) Corresponding image in TEM, showing the granules of the apical compartment (dc), very cohesive and with moderate electron density and denser peripheral portions, and the granules (g), present in the rest of the gland, which are spaced and very heterogeneous in shape and internal patterns. (**D**) Higher magnification of the granules of the apical differentiated compartments. indicating the aspect of moderate and homogeneous electron density (dc). Note the presence of small vesicles (arrowheads) in the matrix of the neighbouring common compartment, indicating possible communication between the different types of syncytial compartments. (M_1_) mucous gland type 1; (cm) cytoplasm matrix.

In some poison glands, we observed another type of syncytial compartment located in the apical region of the gland. Such differentiated compartments are characterized by bearing smaller granules, with a different affinity to toluidine blue when compared to the granules in the common syncytial compartments (Figure 7A). The differences in structure and electron density in the granules and in the cytoplasm of these compartments are evident when the two types of compartments are compared by electron microscopy (Figure 7B–D). In the differentiated apical compartments, the granules are more cohesive and homogeneous and often show borders with more electrodense regions. In addition, the cytoplasm is usually more electron dense, showing clear differences with electron lucent cytoplasm of the common compartments that comprise the rest of the poison glands (Figure 7C,D).

**Figure 8:**
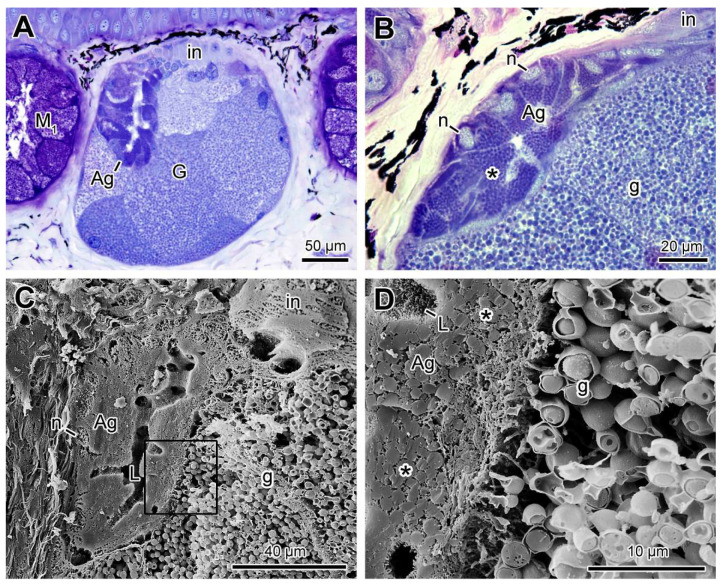
Characterization of the append gland (ag) inside the poison gland of *Siphonops annulatus* (**A**) Section of the poison gland (G) containing the append gland (ag), composed of cells bearing small and aggregated granules, organized to form a lumen. (**B**) High magnification of an append gland (ag) showing its cells with basal nuclei (n) and granules strongly stained by toluidine blue-fuchsin. (**C**) SEM fracture focusing an append gland formed by a set of small cells arranged around a lumen (L). (**D**) Higher magnification of the area delimited in (C), highlighting the interior of the append gland cells (*) and the microvilli facing the lumen (L). (g) Granules of the common syncytial compartments; (in) intercalary zone; (M_1_) mucous gland type 1.

In addition to the internal features already described in the poison glands, in some of them another structure was identified in the upper lateral region, consisting of a set of cells organised to form a discrete lumen and resembling a mucous gland. This lumen seems to connect with the intercalary zone of the poison gland, merging with the main glandular duct (Figure 8A). The cells making up such a structure are mononucleated and contain small, homogeneous granules with a high affinity to toluidine blue (Figure 8A,B). At SEM, the basal nuclei and the homogeneous and compact granules in the cytoplasm are evident, as well as the luminal space with microvilli on the surface (Figure 8C,D). The connection between the ducts is also strongly suggested by the images (Figure 8A,C). Due to the location and the general mucous aspect, we refer this structure as the “append gland” in this work.

**Figure 9:**
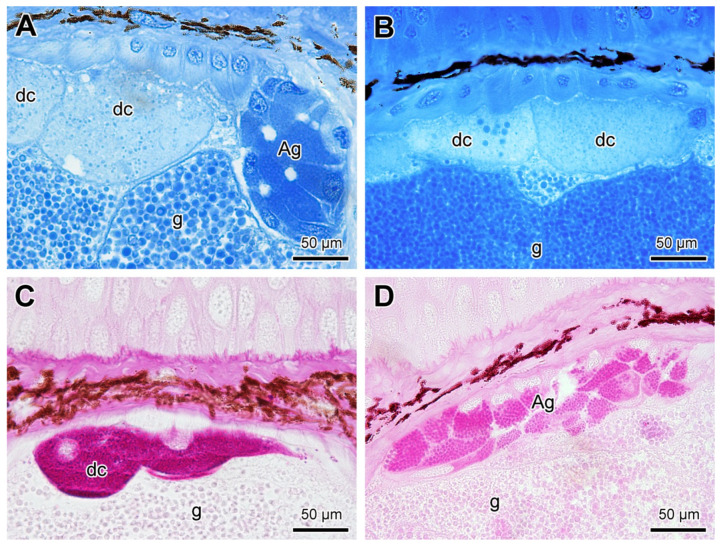
Skin poison glands in *Siphonops annulatus*. (**A**,**B**) bromophenol blue indicating protein content; (**C**,**D**) PAS (periodic acid-Schiff), indicating neutral mucopolysaccharides. (dc) apical differentiated compartments, (ag) append gland, (g) poison granules within the common syncytial compartments.

The common syncytial compartments of the poison glands, regardless of their affinity to toluidine-fuchsin blue, were positive only to bromophenol blue (Figure 9A,B). The differentiated apical compartments, on the other hand, were positive only to the PAS (Figure 9C). Finally, the append gland cells were highly positive both to bromophenol blue (Figure 9A) and to PAS (Figure 9D).

## 3. Discussion

Despite the considerable advances in the study of amphibian skin and skin glands over the last few decades anurans and salamanders always received more attention while caecilians remain least explored. The lack of knowledge of caecilians concerning not only the skin but also several other biological aspects is primarily due to their fossorial habits and consequent difficulty of access, even though some species are relatively abundant in their respective habitats [11,15,37]. Moreover, the restricted distribution of caecilians to tropical and meridional regions historically made caecilians much less studied than more widely distributed anurans and salamanders. These factors possibly contribute to caecilians remaining the least known vertebrate group in most biological aspects [8,11,15].

The skin structure of *Siphonops annulatus* is similar to that of all other amphibians, i.e., it is rich in mucous and poison glands that, despite their epidermal origin, are located in the dermis and easily identified through their morphological and histochemical characteristics [1,2,3,17]. The mucous glands show a typical acinar structure, composed of a cell monolayer delimiting a central lumen and usually positive to mucopolysaccharides. The poison glands are syncytial and full of secretion granules, usually with protein content [2,3,17,26].

Despite the basic structure of the skin mucous and granular glands, each amphibian species shows glands with morphological peculiarities, especially the structure of the secretion granules [23,24]. Moreover, some amphibians show a glandular polymorphism with more than one type of mucous or poison gland [17,23,24,27,28,38,39]. Considering the embryological origin, the morphological differences and similarities among glands may be interpreted as different maturation stages of the same glandular type [23,27,40,41]. In relation to the mucous glands, the comparison of neighboring glands show that their appearance seems to be relatively homogeneous. However, in the poison glands, subtle morphological differences do occur. Moreover, within the same gland, differences in terms of granular density among the various syncytial compartments are usually noted, with compartments at the glandular basis tending to be denser than the superior ones, in the direction of the duct. Based on literature, we believe that such differences may reflex the maturation stages of each compartment [23,27,38].

Based on the acinar structure and the presence of syncytia, the morphological analysis of *Siphonops annulatus* skin showed three different glandular types with typical distribution along the body, two mucous glands and one poison gland [17]. Comparing the mucous glands (M_1_ and M_2_), we revealed significant morphological and histochemical differences. The M_1_ type presents granules with lipid content and flocculent appearance, especially in one of the cell types. Jared and co-authors [17] showed a massive presence of this gland type in the cranial region of *S. annulatus*. In addition to their function in head and body lubrication, helping the animal to access a system of tunnels in a process similar to “diving” into the earth, such glands may also produce toxins able of repelling predators attacking the head underground. When considering defence, it is important to emphasise that in some amphibians the mucous glands are related to the secretion of toxic molecules such as tetrodotoxin [26], one of the most potent neurotoxins in nature [42]. The M_2_ gland, with homogeneous body distribution and a secretion consisting basically of glycoprotein and mucopolysaccharide compounds, may have a role mainly related to the general body moistening, participating in the animal’s gas exchange and homeostasis, similarly to other amphibians [43]. Moreover, skin secretions may provide bacteriostatic and bactericidal action to manage the skin’s microbiota [1,5,6,22,35]. The combination of these factors is evidence of fundamental differences between the two types of mucous gland in *S. annulatus*, rebutting ideas that they can merely represent different stages of the same glandular type. However, studies on the ontogenetic development of the skin glands would be welcome to add new evidence in this respect.

The cutaneous secretion from the poison and mucous glands is understudied among caecilians. In recent decades, our group has been studying the biology and behaviour of *Siphonops annulatus*, particularly in relation to the defence and reproductive behaviour in the field and in captivity [11,17,37,44]. The cacao plantation, located in the south of the State of Bahia (Brazil), where the species is abundant, maintains the original environmental conditions of the Atlantic Rainforest, since cacao trees are planted in the shade of the large original trees (plantation modality known as “cabruca”) [37]. *S. annulatus* remains in the soil, under the thick and moist leaf litter covering the forest floor that forms a constant and favourable mantle and prevents water loss. Our intensive observation of this caecilian over the years allows some considerations about its cutaneous secretion. The animal is highly slippery with intense mucous secretion. Manual manipulation stimulates the release of tiny squirts of secretion that can be observed even with the naked eye. When the animal is handled, the handler (and those close to him) react with sneezing and a runny nose [11].

In moments of defence, the skin secretion, besides mucus, may also contain toxins released from the poison glands. In addition, the accumulation of hypertrophied poison glands at the animal’s rear end was once more confirmed in this work by the examination of three different regions along the body. It seems clear that the tail glands form a type of macrogland precisely in the body region where the animal is most vulnerable [17], reinforcing the role of the poison glands in defence. However, unlike toads [18,24], certain frogs [28], tree frogs [45] and salamanders [20,36,46,47], the macrogland of *Siphonops annulatus* does not form bulges as seen in other amphibians. This may be due to the fossorial environment where a cylindrical and smooth body favours the movement inside the tunnels [17].

Among amphibians the cutaneous poison glands of anurans are best known [1]. Anuran poison glands consist of a large, single syncytium [1,23,24]. In contrast, the poison glands of the gymnophionan *Siphonops annulatus,* as in other caecilian species [2,35,36], are formed by several syncytial units, with peculiar mechanisms of organisation and functioning, which are quite difficult to unravel. As an attempt, we performed several morphological techniques together with a three-dimensional reconstruction, providing essential information and filling such gaps, even if partially. We were able to visualize that *S. annulatus* poison glands consist of a set of juxtaposed syncytial compartments with a low level of cohesion between their external membranes, forming a structure similar to a three-dimensional puzzle. The poison release may consist in the detachment of part of the compartments (or even of entire compartments) through the glandular duct to the skin surface.

Another unusual feature detected in some of the poison glands in *Siphonops annulatus* was the cluster of cells located in a latero-apical position, next to the region that gives rise to the glandular duct (the intercalary region). This cell cluster is mainly constituted of mucous content morphologically resembling a conventional cutaneous mucous gland, with a narrow lumen and apparently connected to the main duct of the poison gland. This unusual structure had already been mentioned in early studies carried out by [48] and, later, by [22], who referred to it as a mixed gland. Due to its odd and puzzling occurrence, these authors considered such structure as a type of “strange body”, forming a physiological and morphological element that might have a role in developing or regenerating the poison gland. Moreover, [22] postulated that such structure, embedded within the poison gland, could be related to poisoning dilution previously to its release through the duct. Here we named this structure the “append gland” due to its location close to the duct and the mucous character of its cells, much resembling the accessory glands observed in toads’ parotoid glands [18,19,49], albeit their external location in relation to the parotoid syncytial units. On the other hand, our data indicate close similarity of such structures with those already observed in salamanders, regarding the location inside the poison gland and the histochemical composition [25,36,39].

In addition to the append gland, we detected some glandular units typically located in apical position in *Siphonops annulatus*, just below the duct, that we termed “differentiated syncytial compartments” due to their syncytial morphology and the granular content essentially consisting of mucus. So far, we have not been able to obtain an indication of a possible function for such glandular units. However, in the salamander *Pleurodeles waltl*, Heiss and co-authors [25] interpreted similar structures as immature cells. A study using *Taricha granulosa* [26] revealed similar structures not consisting of immature secretory units that were proven to be related to the presence of tetrodotoxin.

**Figure 10:**
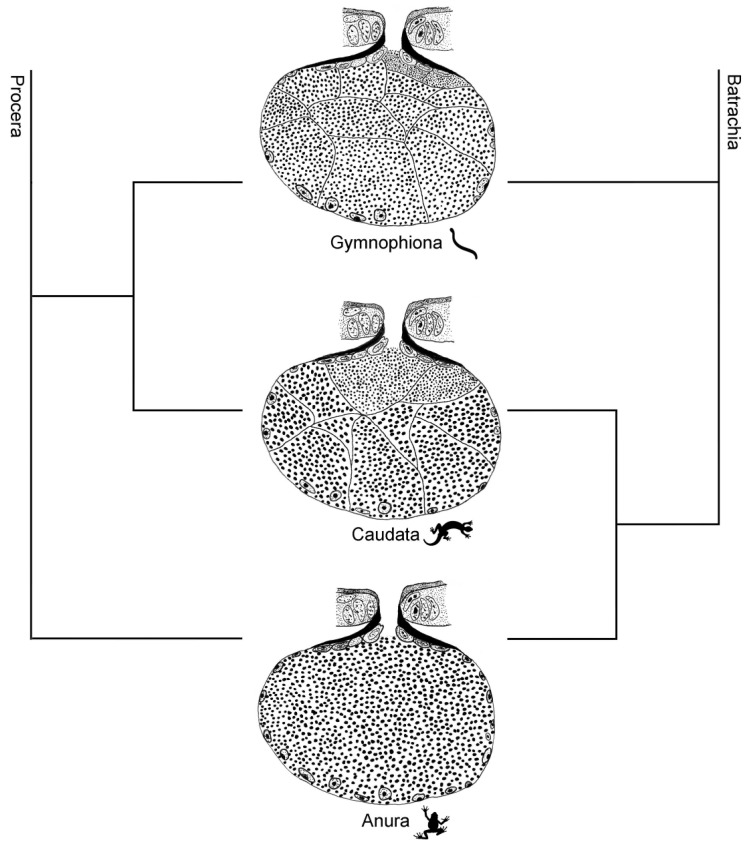
Illustrative scheme showing the morphology of the poison glands in the three orders of extant Amphibia. Note that in caecilians (Gymnophiona) and salamanders (Caudata) the glands are composed of many syncytial compartments while in anurans (Anura) they are composed of a single syncytium. The scheme was based on literature review and personal authors’ observations presented in Table S1.

When the structure of the poison glands is analysed within the context of the different amphibian orders, marked differences can be identified. Concerning complexity, the poison glands of anurans, composed of a single syncytium [23,24,29,38], represent the simplest structure. In contrast, the poison glands of salamanders and caecilians show similarities between them and are formed by several syncytial units (Figure 10) [25,26,27,47]. This pattern was similar even in species of families considered basal in each order, highlighting the structural divergence of the skin poison glands of caecilians and salamanders in relation to anuran amphibians.

Although the phylogenetic affinity between the currently recognised orders of Lissamphibia has been extensively investigated [50,51,52,53,54,55,56,57,58], the relationships between them remain unclear, even though the monophyly of each amphibian order is indisputable [52,53,58,59,60]. Three topologies are recognized: the Batrachia hypothesis, which proposes anurans and salamanders as each other’s closest relatives [3,52,57,58,59,61,62], the Procera hypothesis, suggesting a sister relationship between salamanders and caecilians [51], and Acuda hypothesis [58], which recognizes a clade formed by anurans and caecilians. The Batrachia and Procera hypotheses have received large support among studies [52,53,57,60], although other studies support alternative hypotheses [54,55]. Thus, from the evolutionary point of view, the similarity of the poison glands among caecilians and salamanders corroborates the Procera hypothesis. On the other hand, when considering the Batrachia hypothesis, the total loss of cell membranes by the anuran poison glands may be interpreted as a phylogenetically derived feature.

*Siphonops annulatus* has several cutaneous glandular types that, as reported for several species of amphibian, participate mainly in the chemical defence against predators and microorganisms. Our study shows that, particularly in *S. annulatus*, defence must be very active since the skin contains numerous glands with a characteristic distribution possibly related to its adaptation to the fossorial environment. Although our work makes a valuable contribution to the comparative morphological knowledge of the skin glands of the three orders of amphibian, the functioning mechanism of such structures in caecilians and salamanders remain unknown, especially in respect to the secretory dynamics. Further studies are necessary to understand the mechanism of poison release out of the syncytial structure and posterior glandular refilling process.

## 4. Material and Methods

### 4.1. Animals

Five adult specimens (three females and two males) of *Siphonops annulatus* (Figure 1A) were collected at the experimental farm of the Executive Committee for Cacao Plantation (CEPLAC-CEPEC), municipality of Ilhéus, State of Bahia, Brazil, (SISBIO license #15964-1). The climate is typically humid or sub-humid, with average annual temperatures between 21 °C–25 °C [11]. The animals were maintained in the Structural Biology Laboratory of the Butantan Institute in terraria containing moist soil substrate and coconut half shells on the surface to serve as shelter and fed weekly with earthworms and ground beef or chicken [11].

Selected specimens were euthanised using a lethal dose of sodium thiopental (70 mg/kg) with the addition of Lidocaine (10 mg/mL). 

### 4.2. Histology

Fragments around 1 cm^2^ of dorsal and ventral skin from three body regions (just after the head, mid-body and tail) were collected and fixed for 24–48 h in buffered 4% paraformaldehyde pH 7.2, or Bouin’s solution. The samples were transversely and longitudinally embedded in historesin and paraffin. The material embedded in historesin was sectioned in a semi-automatic microtome Microm^®^ HM340 E (Microm International GmbH part of Thermo Fisher Scientific, Walldorf, Germany) (2 µm–3 µm) and stained with toluidine blue-fuchsin. The samples processed in paraffin were sectioned in the same microtome (5 µm–6 µm), and the sections were stained with haematoxylin-eosin. The sections were also subjected to the following histochemical reactions: bromophenol blue, to indicate the presence of proteins in general, PAS, to identify neutral glycoconjugates, Sudan black B, for detection of lipids, and alcian-blue pH 2.5, for distinguishing acid glycoconjugates [63]. The images were obtained in an Olympus BX51 microscope (Olympus Latin America Inc., Miami, USA) coupled to a digital camera and captured using the CellSens Standard software (Olympus Life and Material Science Europa GMBH, Hamburg, Germany).

### 4.3. Scanning Electron Microscopy (SEM)

Dorsal and ventral skin fragments were fixed in Karnovsky [64] fixative solution (5% glutaraldehyde + 4% paraformaldehyde, in 0.1 M cacodylate buffer, pH 7.2), for 24 h. After washings in cacodylate buffer, the fragments were post-fixed in 1% osmium tetroxide in cacodylate buffer. Additionally, some of the fixed fragments were submerged in dimethyl sulfoxide (DMSO), frozen in liquid nitrogen and fractured with the aid of a frozen razor blade. The fractured pieces were then dehydrated in ethanol crescent series, dried in a critical point device, mounted on aluminium stubs, covered with gold in a sputtering apparatus, and examined using a FEI Quanta 250 scanning microscope, operating at 10 kV.

### 4.4. Transmission Electron Microscopy (TEM)

Dorsal and ventral skin fragments were fixed in Karnovsky [64] solution for 24 h. After washing in the same buffer, the samples were post-fixed in 1% osmium tetroxide in cacodylate buffer, dehydrated in ethanol crescent series, and embedded in epoxy resin (Polybed, Electron Microscopy Sciences). Ultrafine sections (60 nm) were contrasted in 2% uranyl acetate and lead citrate and examined using a LEO 906E transmission electron microscope, operating at 80 kV.

### 4.5. Three-Dimensional Reconstruction of the Poison Gland

The 3D reconstruction was performed using the free software Reconstruct [65]. After aligning the images obtained from serial histological sections (6 µm) of dorsal skin, the cell compartments comprising the poison glands were manually delimited in each of the images. The composition of all lines generated from the cells boundaries was then used to reconstruct the three-dimensional structure.

## Data Availability

Data are available upon request; please contact the contributing authors.

## Supplementary Materials

The following are available online at https://www.mdpi.com/article/10.3390/toxins13110779/s1, Table S1: Literature review and personal authors’ observations about the skin glands in the three amphibian orders, giving support to the scheme presented in Figure 10 (Table S1), Video S1: Three-dimensional reconstruction of the poison gland obtained from aligned histological sections (Video S1).
