# Supplementary material for: Morphology of the Cutaneous Poison and Mucous Glands in Amphibians with Particular Emphasis on Caecilians (Siphonops annulatus)
Source: Toxins (Basel). 2021 Nov 4;13(11):779. doi: 10.3390/toxins13110779 (PMC8617868; doi:10.3390/toxins13110779)
Supplement: Supplementary file 1 [file toxins-13-00779-s001.zip › Mauricio et al - Table S1.pdf]

ORDER	FAMILY	SPECIES	AUTHOR	REFERENCE
Anura	Bombinatoridae	*Bombina orientalis*	Quagliata et al., 2008	https://doi.org/10.13128/Acta_Herpetol-2680
	Bufonidae	*Rhinella marina e Rhaebo guttatus*	Mailho-Fontana et al., 2014	https://doi.org/10.1002/jez.1838
	Bufonidae	*Rhinella marina*	Mailho-Fontana et al., 2018	https://doi.org/10.1186/s12983-018-0294-5
	Bufonidae	*Rhinella icterica*	Regis-Alves et al., 2017	https://doi.org/10.1016/j.toxicon.2017.07.022
	Bufonidae	*Rhinella arenarum*	Regueira et al., 2015	https://doi.org/10.1002/ar.23284
	Bufonidae	*Ollotis nebulifer e Anaxyrus speciosus*	Powell et al., 2008	https://doi.org/10.1002/ar.23284
	Bufonidae	*Melanophryniscus stelzneri*	Delfino et al.,1998	https://doi.org/10.1002/(sici)1097-4687(199807)237:1%3C19::aid-jmor2%3E3.0.co;2-j
	Bufonidae	*Rhinella granulosa*	Delfino et al.,1999	https://doi.org/10.1016/s0041-0101(98)00267-0
	Bufonidae	*Rhinella jimi*	Jared et al., 2009	https://doi.org/10.1016/j.toxicon.2009.03.029
	Cycloramphidae	*Cycloramphus fuliginosus*	Gonçalves and Brito-Gitirana, 2007	http://dx.doi.org/10.1016/j.micron.2007.08.005
	Odontophrynidae	*Odontophrynus cultripes*	Mailho-Fontana et al., 2017	https://doi.org/10.1016/j.toxicon.2017.02.022
	Dendrobatidae	*Dendrobates auratus*	Angel et al., 2002	https://doi.org/10.1016/s0041-0101(02)00206-4
	Dendrobatidae	*Phyllobates bicolor*	Delfino et al., 2010	http://dx.doi.org/10.1643/CG-08-134
	Hylidae	*Phyllomedusa hypocondrialis.*	Delfino et al., 1998a	https://doi.org/10.1016/S0040-8166(98)80004-9
	Hylidae	*Phyllomedusa distincta*	Antoniazzi et al., 2013	https://doi.org/10.1111/jzo.12044
	Hylidae	*Hyla regilla*	Delfino et al 2006	https://doi.org/10.1016/j.tice.2005.11.002
	Hylidae	*Phyllomedusa hypochondrialis*	Delfino et al.,1997	https://doi.org/10.1016/s0040-8166(98)80004-9
	Hylidae	*Hyla regilla*	Delfino et al.,2005	https://doi.org/10.1016/j.tice.2005.11.002
	Hylidae	*Hypsiboas pulchellus*	Brunetti et al., 2016	https://doi.org/10.1111/joa.12413
	Hylidae	*Ololygon* and *Scinax*	Silva et al., 2017	https://doi.org/10.3897/zoologia.34.e20176
	Hylidae	*Phyllomedusa hypochondrialis azurea*	Nosi et al., 2002	http://dx.doi.org/10.1007/s004350100051
	Leiopelmatidae	*Leiopelma* spp	Melzer et al., 2011	https://doi.org/10.1002/jmor.10960
	Leiuperidae	*Pleurodema and Somuncuria*	Ferraro et al., 2011	https://doi.org/10.1111/j.1463-6395.2011.00529.x
	Leptodactylidae	*Engystomops pustulosus*	Delfino et al., 2015	https://doi.org/10.1002/ar.23189
	Leptodactylidae	*Physalaemus nattereri*	Lenzi-Mattos et al., 2005	https://doi.org/10.1017/S095283690500703X
	Ranidae	*Rana cancrivora*	Seki et al., 1995	https://doi.org/10.2108/zsj.12.623
	Ranidae	*Niridia pleuraden*	Gong et al., 2020	https://doi.org/10.1186/s40851-020-00160-w
	Ranidae	*Clinotarsus curtipes*	Gosavi et al., 2014	http://dx.doi.org/10.1016/j.cbpa.2014.01.009
	Telmatobiidae	*Telmatobius* spp	Barrionuevo, 2015	https://doi.org/10.1111/joa.12413

ORDER	FAMILY	SPECIES	AUTHOR	REFERENCE
Gymnophiona	Caeciliidae	*Gegeneophis ramaswamii*	Arun et al., 2019	https://doi.org/10.1002/jemt.23276
	Caeciliidae	*Ichthyophis tricolor and Uraeotyphlus cf. oxyurus,*	Arun et al., 2018	https://doi.org/10.1016/j.micron.2018.01.004
	Ichthyophiidae	*Ichthyophis orfhoplicafus e Ichthyophis kohtaoensis*	Fox, 1983	https://doi.org/10.1111/j.1469-7998.1983.tb02092.x
	Rhinatrematidae	*Rhinatrema* sp	Mailho-Fontana, pers. communication	-
	Siphonopidiae	*Siphonops annulatus*	Jared et al., 1999	https://doi.org/10.1016/S1095-6433(99)00076-8
	Siphonopidiae	*Siphonops annulatus*	Sawaya,1938	https://doi.org/10.11606/issn.2526-4877.bsffclzoologia.1938.113915
	Siphonopidiae	*Siphonops annulatus*	Jared et al., 2018	https://doi.org/10.1038/s41598-018-22005-5
Caudata	Hinobidae	*Hynobius retardatus*	Ohmura and Wakahara, 1998	https://doi.org/10.1046/j.1432-0436.1998.6350237.x
	Hinobidae	*Batrachuperus pinchonii* and *Hynobius chinensis*	Xiong et al., 2013	https://doi.org/10.3724/sp.j.1245.2013.00062
	Plethodontidae	Aneides lugubris	Staub and Paladin, 1997	https://www.researchgate.net/publication/287854489
	Plethodontidae	*Karsenia koreana*	Sever et al., 2016	https://doi.org/10.1643/CG-16-468
	Plethodontidae	*Plethodon cinereus*	Sever and Siegel, 2015	https://doi.org/10.1002/jmor.20342
	Salamandridae	*Ambystoma gracile*	Brodie and Gibson, 1969	https://www.jstor.org/stable/3891393
	Salamandridae	*Taricha granulosa*	Hippe et al., 2014	https://doi.org/10.1643/CH-13-080
	Salamandridae	*Pleurodeles waltl*	Heiss, et al., 2009	https://doi.org/10.1002/jmor.10728
	Salamandridae	*Plethodon shermani*	Von Byern et al., 2015	https://doi.org/10.1016/j.zool.2015.04.003
	Salamandridae	*Plethodon shermani*	Largen and Woodley, 2008	https://doi.org/10.1655/08-010.1
	Salamandridae	*Tylototriton verrucosus*	Wanninger et al., 2018	https://doi.org/10.1186/s40851-018-0095-x
	Salamandridae	*Lyciasalamandra billae* and *Lyciasalamandra luschani basoglui*	Akat et al., 2014	http://dx.doi.org/10.2478/s11756-013-0313-0
	Salamandridae	*Desmognathus fuscus*	Mcmanus, 1935	https://www.jstor.org/stable/24332083?seq=1
	Salamandridae	*Ensatina eschscholtzii*	Fontana et al., 2006	https://doi.org/10.1111/j.1095-8312.2006.00592.x
	Salamandridae	*Salamandra Luschani*	Staub et al., 2005	https://doi.org/10.1655/04-84.1
	Salamandridae	*Mertensiella caucasica e M. luschani*	Sever et al., 1997	https://doi.org/10.1002/(SICI)1097-4687(199704)232:1<93::AID-JMOR6>3.0.CO;2-P
	Salamandridae	*Taricha granulosa*	Mailho-Fontana et al., 2019	https://doi.org/10.1038/s41598-019-54765-z
	Salamandridae	*Cynops pyrrhogaster*	Tsuruda, 2001	https://doi.org/10.1016/s0041-0101(01)00198-2
	Salamandridae	*Ambystoma gracile*	Licht and Sever, 1993	http://dx.doi.org/10.2307/1446302
	Salamandridae	*Salamandra salamandra*	Brodie and Smatresk, 1990	https://www.jstor.org/stable/38925955
	Salamandridae	*Triturus karelinii*	Bingol-Ozakpinar and Murathanoglu, 2011	http://dx.doi.org/10.2478/s11756-011-0009-2
	Sirenidae	*Siren intermedia*	Reno and Middleton III, 1973	https://doi.org/10.1111/j.1463-6395.1973.tb00440.x
